# Assessing VirScan serosurvey epitope profiling variability between in-clinic venous blood draw and capillary blood self-sampling device

**DOI:** 10.1128/spectrum.02454-25

**Published:** 2026-04-27

**Authors:** Linda M. Sircy, Terry L. Stevens-Ayers, Elizabeth M. Krantz, Laurel Joncas-Schronce, Nina S. O. Ozbek, Rachel L. Blazevic, Larry Mose, Louise E. Kimball, Ryan Basom, Sayan Dasgupta, Antje Heit, Frank Schmitz, David Heckerman, Rachel A. Bender Ignacio, Joshua A. Hill, Jim Boonyaratanakornkit, Michael Boeckh, Alpana Waghmare

**Affiliations:** 1 Vaccine and Infectious Disease Division, Fred Hutchinson Cancer Center 7286, Seattle, Washington, USA; 2 Clinical Research Division, Fred Hutchinson Cancer Center 7286, Seattle, Washington, USA; 3 Amazon, Seattle, Washington, USA; 4 Department of Pediatrics, University of Washington 271845, Seattle, Washington, USA; 5 Seattle Children’s Research Institute, Seattle, Washington, USA; Barnard College, Columbia University, New York, New York, USA

**Keywords:** VirScan, antibody profiling, blood self-sampling, epitope profiling

## Abstract

Timely and ongoing in-clinic sample collections are a common logistical barrier to volunteer participation and retention in longitudinal clinical studies. To remove this barrier, clinical studies have recently begun to implement the use of at-home capillary blood self-sampling devices in place of in-clinic venous blood draws for participant blood sample collection. Thus, we assessed antibody responses to a broad library of over 300 viral, bacterial, and fungal pathogens using VirScan immunoassay testing on adult volunteer capillary (serum) and venous (plasma) blood samples collected by Tasso devices and in-clinic venipunctures on the same day to determine whether self-sampling devices are a more practical and convenient alternative for future clinical studies. Most VirScan measurements of antibodies specific for clinically relevant respiratory viruses and herpesviruses had strong concordance between participant-matched Tasso and venipuncture blood samples. While we did not identify a systematic bias in most pathogen-specific antibody measurements associated with blood collection method, we did observe intrinsic VirScan inter-assay variability across some clinically relevant viruses. Together, our data demonstrate that capillary blood self-sampling devices are a practical alternative to in-clinic venipunctures for VirScan clinical research studies. However, these blood collection methods should not be used interchangeably within longitudinal studies to minimize the introduction of technical variables.

**IMPORTANCE:**

Our study assessed whether capillary blood self-sampling devices could reliably replace in-clinic venous blood collection methods for the VirScan immunoassay, which can detect antibodies specific to hundreds of pathogens. Longitudinal studies requiring multiple in-clinic visits for sample collection often experience low volunteer retention because of the inconvenience of traveling to research sites. Allowing volunteers to use at-home self-sampling devices reduces the burden of travel for participants and increases access to outreach for volunteers who would otherwise not participate in research. Importantly, VirScan only requires a small sample volume, so blood self-sampling devices would be appropriate to use despite their volume collection limitations. Overall, capillary blood self-sampling devices can be a reliable and efficient method for research studies to investigate antibody responses longitudinally using VirScan. However, to limit the introduction of technical variables, collection methods should not be used interchangeably within a longitudinal study.

## INTRODUCTION

Longitudinal prospective clinical studies often face low rates of recruitment and declining retention over time (1–4). Since participant attrition often increases throughout a study period, prospective studies requesting multiple clinical site visits for biological sample collections may experience intermittent and reduced sample collection over time. In recent years, home-based capillary blood self-collection device usage has increased in clinical research studies as an alternative to in-clinic venous blood draws. Multiple clinical studies have found overwhelmingly positive feedback from participants on the use of blood self-sampling devices, specifically noting the ease of use, minimal pain caused by the device, and the preference to collect samples at home over travel to clinical sites for blood draws (5–8). Furthermore, whole blood and dried blood specimens collected with self-sampling devices compared to venipuncture blood samples show strongly correlated results for HIV pre-exposure prophylaxis monitoring (7), detection of cytomegalovirus DNAemia (8), autoantibodies and inflammatory markers in patients with immune-mediated rheumatic diseases (9, 10), biomarkers and analytes from blood (11–16), and anti-SARS-CoV-2 antibodies (16–20). However, whether blood self-sampling devices can be used in place of venipuncture blood collection has not been widely tested for downstream applications with high-throughput next-generation sequencing assays.

Viral epitope scanning (VirScan) is a high-throughput phage-immunoprecipitation sequencing (PhIP-Seq) antibody survey assay that utilizes a bacteriophage display library of over 113,000 unique synthetic linear peptide epitopes spanning over 450 organisms, including viruses, bacteria, fungi, parasites, and allergens (21). The VirScan assay requires as little as 2 μg of immunoglobulin protein per sample, which equates to less than 5 μL of serum or plasma from healthy donors. The Tasso blood self-collection kits can collect up to 600 μL of whole blood, providing sufficient serum immunoglobulin concentration for VirScan testing.

In this study, we assessed historical VirScan data used to measure antibodies specific for clinically relevant viruses and bacteria in a convenience sampling of participant-matched paired capillary (serum) and venous (plasma) blood samples collected by Tasso self-sampling devices and in-clinic venipunctures. The goal of this study was to determine if blood self-sampling devices are a suitable blood collection method for VirScan immunoassay testing and a practical alternative to in-clinic venipuncture blood collection methods.

## MATERIALS AND METHODS

### Participant samples

We conducted a longitudinal prospective study between April 2020 and June 2021 at Fred Hutchinson Cancer Center (Fred Hutch), following healthy adults (≥18 years) at increased risk of exposure to SARS-CoV-2. Participants had venous blood draws at baseline, at 28 days post-SARS-CoV-2 infection, and at the end of the study. Participants mailed in samples self-collected by Tasso (Tasso, Inc.) devices monthly and changing to weekly following SARS-CoV-2 infection. We identified convenience samples from 34 study participants with paired samples of blood collected by the Tasso self-sampling device and from venipuncture collected on the same day, totaling 36 participant-matched paired blood samples. Two participants had timely paired blood samples at two time points.

### Blood sample collection and processing

Whole blood samples collected by venipuncture performed at an on-site clinic were collected into Acid Citrate Dextrose-containing vacutainer tubes and processed in our laboratory within 8 hours of the procedure. Plasma was separated from the cellular component by centrifugation. Capillary blood was collected by the Tasso self-sampling device attached to the upper arm into a collection tube with a gel separator. Serum was separated from the blood clot layer by centrifugation. Tasso device collections were either performed at an on-site clinic or at the volunteer’s home and mailed to our laboratory; thus, some samples were delayed in receiving and processing. For Tasso-collected blood samples, 30 samples were processed within 8 hours of collection, four samples were processed between 23 and 73 hours after collection, and two samples were processed over 100 hours after collection. Serum samples from one additional healthy adult control donor independent from the longitudinal study were also assessed by VirScan. For the control donor, whole blood was collected by venipuncture at a single time point, and serum was separated from the cellular component by centrifugation. All plasma and serum sample aliquots were stored at −80°C. For the healthy control donor, serum aliquots were kept frozen until use and did not undergo multiple freeze-thaws. The VirScan assay data evaluated in this study originate from whole blood samples collected in compliance with the original Institutional Review Board-approved study protocol completed in 2021 and represent a convenience sample of historical data.

### Viral epitope scanning (VirScan) assay

The VirScan assay was carried out with combined T7 bacteriophage Vir3 and modified coronavirus-specific libraries (22, 23). The VirScan phage library batches used were produced in January 2021 (CoV) and March 2021 (Vir3). Serosurvey of IgG antibodies from serum and plasma samples in technical duplicates was conducted with the VirScan assay, as previously described (21, 23–25).

Serum and plasma samples were diluted 1:10 with phosphate-buffered saline for phage-antibody complex formation. Plates were either arranged with technical sample duplicates on separate plates (VS7–VS13) or on the same plate (VS21–VS28). Serum and plasma samples from individual participants were sequenced together when possible, excluding sequencing of repeat samples necessary for samples with poor sequencing quality. DNA sequencing libraries were prepared with a fragment insert size of 376 bp, with two batches of 192 7-bp barcodes used per sequencing batch. Libraries were pooled and sequenced on the Illumina HiSeq platform using single-read sequencing and 50-cycle runs, yielding approximately 750,000 reads (mean/median) per barcode (minimum 400,000, maximum 1.1 million). Because of the requirement for custom sequencing primers, two sequencing batches were run in one flow cell each of a two-lane Illumina chip.

Final read depth for the samples included in this study for the Vir3 phage library samples was a median of 7.9, mean of 7.9, and range of 4.5–11. Final read depth for the samples included in this study for the CoV phage library samples was a median of 9.1, mean of 11, and range of 4–48.6. Percent reads aligned to the Vir3 library were a median of 77.7%, mean of 75.7%, and range of 44.6%–82.3%. Percent reads aligned to the CoV library were a median of 5.2%, mean of 7.2% and range of 1.6%–37.5%.

DNA sequences were processed and aligned to reference sequences using the Nextflow pipeline PhIP-Flow (version 1.14) of the phippery software suite (26). Determination of positive detection of antibody-specific epitope binding was adapted from Mina et al. (2019) using a minimum Z-score threshold of 7 (27). Binning for all samples in this study was determined using 10 randomly selected mock IP (bead only) wells from across plates. Other phippery input parameters included read length set to 50, peptide length set to 50, and number of mismatches allowed set to 2. The average of both duplicate sample Z-scores for each peptide is calculated and reported as the epitope-binding signal (EBS) score and is a measure of antibody abundance to one peptide and analogous to a titer. Summary statistics for each organism were calculated, including: (i) total epitope hits, which is the sum of all positively detected antibody-bound peptides; and (ii) geometric mean (gMean) EBS scores of all positively detected peptide hits. Total epitope hits and gMean EBS scores for each organism were calculated after exclusion of peptides with lower EBS scores that overlap another peptide by ≥7 amino acids with the higher EBS score, as previously described (21).

The coronavirus group included the human coronaviruses (HCoV), 229E, OC43, NL63, HKU1, and SARS-CoV-2. HCoV-HKU1 VirScan library peptides are separated into one non-specific HKU1 entry and three separate isolates (N1, N2, N5). Out of 1,375 unique peptides across the four HCoV-HKU1 VirScan phage library entries, only 41 (3%) are duplicated across two or more strains. In this study, only one peptide in one sample was found duplicated in the HCoV-HKU1 N2 and N5 isolates; thus, all four HCoV-HKU1 organisms were included in our analyses. The rhinovirus group included Rhinovirus A and B species. Two pneumoviruses, respiratory syncytial virus (RSV) and human metapneumovirus (HMPV), were analyzed with parainfluenza virus (PIV) types 1–4 and influenza (flu) A and B. The human herpesvirus (HHV) group included herpes simplex viruses 1 (HSV1) and 2 (HSV2), varicella-zoster virus, Epstein-Barr virus (EBV), cytomegalovirus (CMV), HHV6A, HHV6B, HHV7, and HHV8. The human adenovirus group included adenoviruses A–F species. The bacterial group included *Staphylococcus aureus* and *Streptococcus pneumoniae*.

### Statistical analyses

Data processing and statistical analysis were performed in R (version 4.4.3) (28) and RStudio (version 2024.12.1+563) (29) using base R and rstatix (30), epiR (31) and yardstick (32) for calculating Lin’s concordance correlation coefficients (CCC) (33), and rmarkdown (34–36), knitr (37–39), kableExtra (40), gridExtra (41), and the tidyverse suite (42) for tables and figures. For Bland-Altman (43) analyses, 95th percentile center range was calculated instead of limits of agreement due to the non-normal distribution of data. Bland-Altman analyses of epitope hits and gMean EBS scores for all viruses, bacteria, and fungi/yeast organisms were conducted both including and excluding paired capillary and venous blood samples with zero epitope hits (zero-zero pairs). An additional sensitivity analysis was performed by evaluating median difference and mean absolute deviation (MAD) to assess the impact of including zero-zero pairs.

CCC were interpreted similarly to Altman (44), but with fewer categories (less than 0.2 as poor, 0.2–0.49 as weak, 0.5–0.79 as moderate, and 0.8 or greater as strong). CCC 95% confidence intervals were calculated using bias-corrected and accelerated bootstrap methodology (1,000 iterations) and assumed all 36 sets of paired measurements from the 34 individuals were independent. Because we might expect correlation among sets of paired measurements belonging to the same individual (two individuals contributed two pairs each), we conducted a sensitivity analysis in which we selected only one pair per individual for the calculation of CCC and corresponding confidence intervals.

To best assess inter-assay variability, reproducibility, and concordance between participant-matched blood samples, anomalous outliers were not excluded from data analyses. For the coefficient of variation calculation for each organism, first the mean of gMean EBS scores within each of three independent assay runs was calculated. Second, the mean and standard deviation were calculated for each organism from the three independent assays’ means of gMean EBS scores. Finally, the coefficient of variation, standard deviation divided by the mean, was calculated and reported as a percentage.

## RESULTS

Between April 2020 and June 2021, we conducted a longitudinal prospective study following healthy adult volunteers with a high risk of exposure to SARS-CoV-2. Within the observation period, we identified convenience samples from 34 study participants with blood samples collected by Tasso self-sampling device and venipuncture on the same day, totaling 36 pairs of participant-matched capillary (serum) and venous (plasma) blood samples. We performed VirScan epitope profiling to measure antibody responses to a broad library of pathogens, to assess inter-assay variability and reproducibility, and to identify whether blood collection method affects assay results.

**Fig 1:**
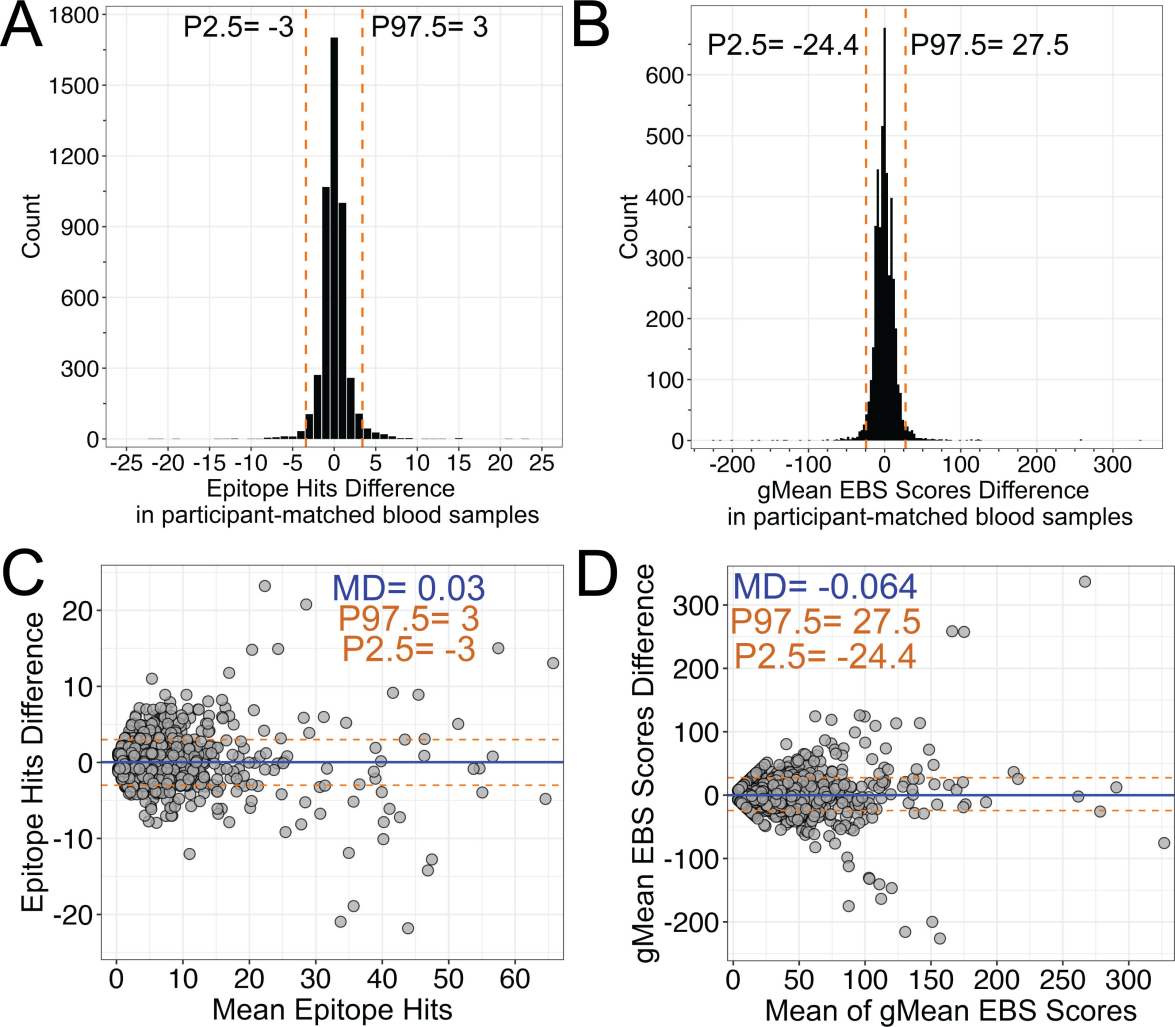
VirScan analysis of antibody responses to viral, bacterial, and fungal organisms in participant-matched Tasso and venous blood draw samples. (**A, B**) Histogram of differences in (**A**) epitope hits and (**B**) gMean EBS scores for all viral, bacterial, and fungal organisms. Orange dashed lines represent the 2.5–97.5th interpercentile range (P2.5, P97.5) with values indicated. For panel **B**, bin width equals 3. (**C, D**) Bland-Altman analysis of (**C**) epitope hits and (**D**) gMean EBS scores for all viral, bacterial, and fungal organisms. Orange dashed lines represent the 2.5–97.5th interpercentile range with values indicated. Blue solid line represents the mean of the differences (MD) with values indicated. Organisms with no hits in both participant-matched Tasso and blood draw samples (zero-zero pairs) were excluded.

We first assessed the variability of total epitope hits and gMean EBS scores between participant-matched Tasso serum and venipuncture plasma blood samples for all viral, bacterial, and fungal organisms included in the VirScan phage libraries (Fig. 1). Excluding the data from organisms with zero epitope hits in both Tasso and venipuncture participant samples (zero-zero pairs) (*n* = 7,426), 95% of epitope hit differences in paired blood samples ranged between ±3, while 95% of gMean EBS scores ranged between −24.4 and 27.5 (*n* = 4,705) (Fig. 1A and B). We then measured the agreement of the same VirScan metrics between participant-matched capillary and venous blood samples using the Bland-Altman method, which plots the difference between two measurements against their mean. A mean difference of zero within Bland-Altman analyses indicates no difference (or no bias) between the two measurements. Our data suggest that both epitope hits and gMean EBS scores had no systematic bias for either Tasso serum or venipuncture plasma samples, as the mean differences between participant-matched samples were 0.03 for epitope hits and −0.064 for gMean EBS scores (Fig. 1C and D). Excluding zero-zero pairs yielded a median difference of 0 and mean absolute deviation (MAD) of 1 for epitope hits, and a median difference of −0.567 and MAD of 7.735 for gMean EBS scores (Table S1). Zero-zero pairs were excluded from analysis because their inclusion biased both the mean difference and 95th percentile center range to be closer in agreement for both epitope hits and gMean EBS scores (Table S1). Both median difference and MAD estimates also indicated that agreement conclusions were sensitive to the inclusion of zero-zero observations (Table S1). Together, these data demonstrate a high degree of agreement between epitope hits and gMean EBS scores between participant-matched capillary (serum) and venous (plasma) blood samples, but also indicate some assay variability when assessing antibody responses to the broader library of pathogens by VirScan.

**Fig 2:**
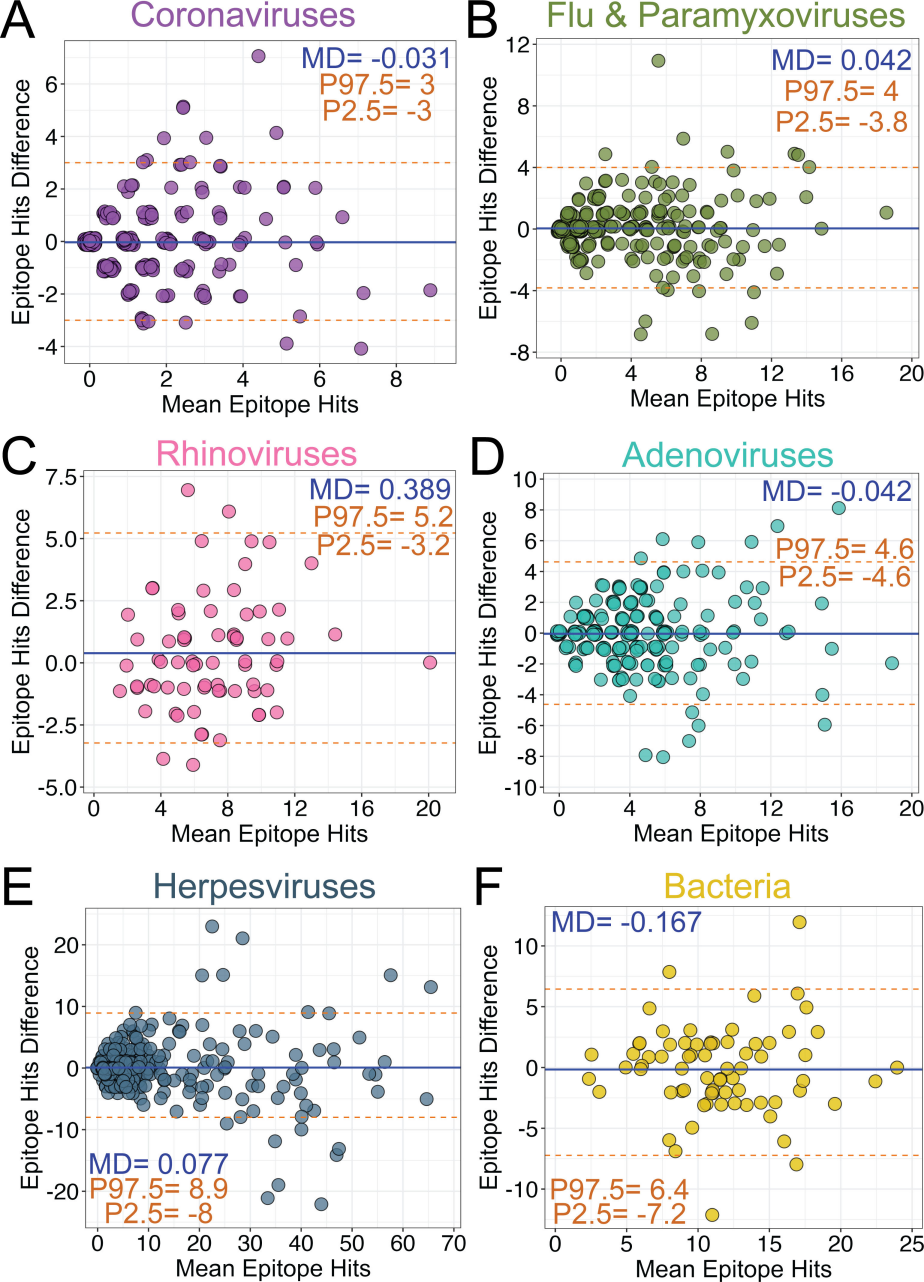
VirScan analysis of total antibody-bound epitope hits of clinically relevant viruses and bacteria in participant-matched Tasso and venous blood draw samples. (**A–D**) Bland-Altman analysis of epitope hits in (**A**) human coronaviruses, (**B**) influenza and clinically relevant respiratory paramyxoviruses, (**C**) rhinoviruses A and B, (**D**) adenoviruses, (**E**) herpesviruses, and (**F**) bacteria (*S. aureus, S. pneumoniae*). Orange dashed lines represent the 2.5–97.5th interpercentile range (P2.5, P97.5) with values indicated. Blue solid line represents the MD with values indicated.

**Fig 3:**
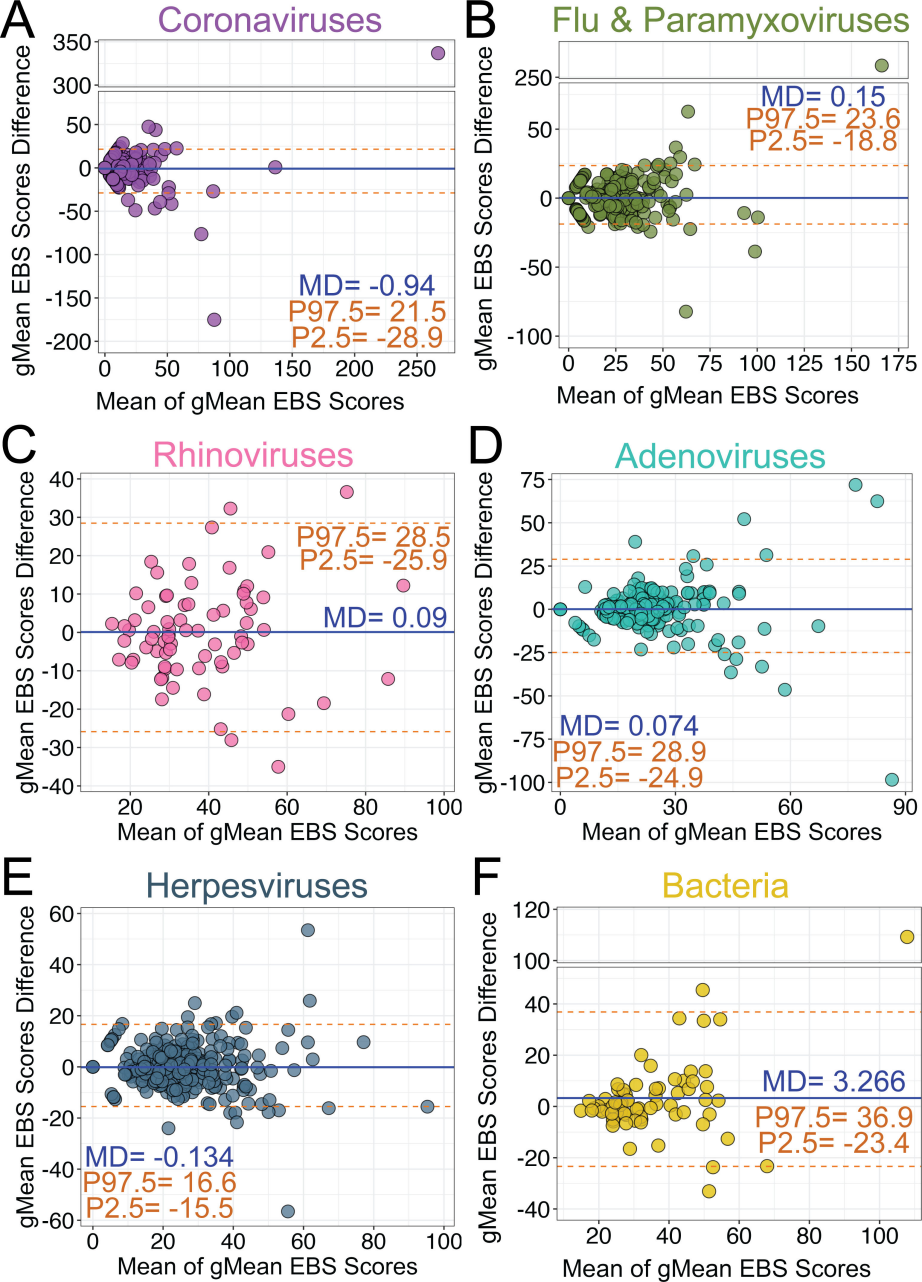
VirScan analysis of antibody abundance to epitopes of clinically relevant viruses and bacteria in participant-matched Tasso and venous blood draw samples. (**A–D**) Bland-Altman analysis of the gMean EBS scores in (**A**) human coronaviruses, (**B**) influenza and clinically relevant respiratory paramyxoviruses, (**C**) rhinoviruses A and B, (**D**) adenoviruses, (**E**) herpesviruses, and (**F**) bacteria (*S. aureus, S. pneumoniae*). Orange dashed lines represent the 2.5–97.5th interpercentile range (P2.5, P97.5) with values indicated. Blue solid line represents the MD with values indicated.

We next focused our investigation on whether there were differences in VirScan measurements of antibody responses to 33 clinically relevant respiratory viruses and herpesviruses and 2 bacterial species between participant-matched capillary and venous blood samples. We assessed agreement of epitope hits (Fig. 2) and gMean EBS scores (Fig. 3) between participant-matched blood samples using the Bland-Altman method for smaller groups of clinically relevant viruses and bacteria. For epitope hits, the mean differences across all pathogen groups assessed ranged from −0.167 (bacteria) (Fig. 2F) to 0.389 (rhinoviruses) (Fig. 2C). For gMean EBS scores, the mean differences for the groups of viruses ranged from −0.94 (coronaviruses) (Fig. 3A) to 0.15 (influenza and paramyxoviruses) (Fig. 3B). Together, these data demonstrate an overall lack of systematic measurement bias associated with blood collection method or blood sample type for clinically relevant viruses. However, *S. aureus* and *S. pneumoniae* had a higher bias (3.266) toward the venipuncture plasma blood samples, though this was primarily driven by one outlying sample (Fig. 3F). For the four respiratory viral groups, the 95th percentile center range for epitope hit differences between paired blood samples was smallest for the coronavirus group (−3 to 3) and largest for the adenovirus group (−4.6 to 4.6) (Fig. 2A through D). The herpesvirus group had the largest 95th percentile center range, from −8 to 8.9 (Fig. 2E), while the 95th percentile center range for *S. aureus* and *S. pneumoniae* was between −7.2 and 6.4 (Fig. 2F). However, the herpesvirus group had the smallest 95th percentile center range for differences in gMean EBS scores, ranging from −15.5 to 16.6 (Fig. 3E) compared to the other pathogen groups assessed. These data suggest that while total epitope hits for herpesviruses can be more variable in paired blood samples, the gMean EBS scores, which are a measure of antibody abundance, are more comparable. In addition, the Bland-Altman analyses show that as the mean of gMean EBS scores of paired blood samples increased, the differences also increased (Fig. 3). These data suggest that as antibody abundance measurements increase, the agreement between paired sample measurements is diminished. However, for participant samples with weaker agreements between paired Tasso serum and venipuncture plasma sample measurements, we found no association between larger differences in measurements compared to either low whole blood volumes collected by Tasso devices (Fig. S1A and B) or delays in time from sample collection to processing in the laboratory (Fig. S1C and D).

**Fig 4:**
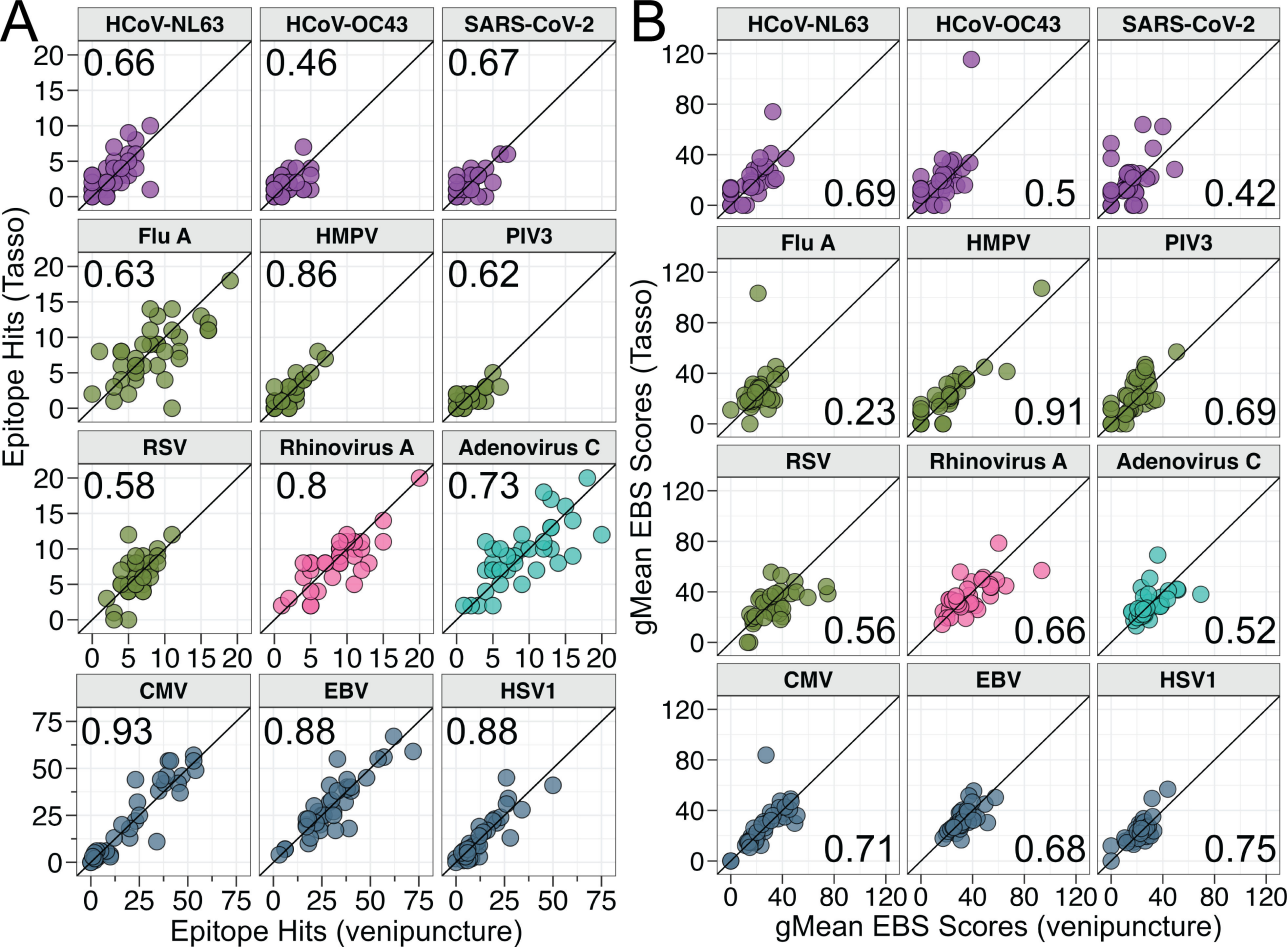
Concordance analysis of antibody responses measured by VirScan to clinically relevant respiratory viruses and herpesviruses in participant-matched Tasso and venous blood draw samples. (**A, B**) Scatter plots comparing participant-matched Tasso and venipuncture blood samples for (**A**) epitope hits and (**B**) gMean EBS scores for select clinically relevant viruses. Black solid line represents an identity reference line (y = x). Values indicated are concordance correlation coefficients.

We next investigated the concordance of epitope hits and gMean EBS score measurements for participant-matched blood samples by performing CCC calculations for pathogen groups and individual organisms. For epitope hits, all pathogen groups had either moderately (>0.5) or strongly (>0.8) concordant measurements between participant-matched blood samples (Table S2). However, the bacteria and coronavirus groups had weakly (<0.5) concordant gMean EBS scores between participant-matched blood samples (Table S2). The herpesvirus group had the most concordant epitope hits (0.95) and gMean EBS scores (0.80) (Table S2) between participant-matched blood samples. We then assessed concordance between epitope hits and gMean EBS scores for individual viruses and bacteria to identify whether specific organisms were driving discordance between blood samples. The concordance between epitope hits (Fig. 4A; Table S2) and gMean EBS scores (Fig. 4B; Table S2) between blood samples for respiratory viruses varied from poorly (<0.2) concordant to strongly concordant. However, as expected from the strong concordance of measurements within the entire group, all individual herpesvirus epitope hits (Fig. 4A; Table S2) and gMean EBS scores (Fig. 4B; Table S2) were either moderately or strongly concordant between participant-matched blood samples. These data suggest that the larger 95th percentile center range for herpesviruses’ epitope hits (Fig. 2E) compared to the respiratory viruses was not primarily due to discordance of measurements between participant-matched blood samples. In addition, our data suggest that the repeated measures for two of the participants included within the analyses had little effect on the overall concordance between epitope hits (Table S3) and gMean EBS scores (Table S4) for most of the pathogens evaluated. For epitope hits, the categorical interpretation after removing the repeated samples did not change for any pathogen (Table S3). For gMean EBS scores, HHV6B was the only pathogen where the categorical interpretation was reduced to a weaker category after removing the repeated measures (Table S4).

**Fig 5:**
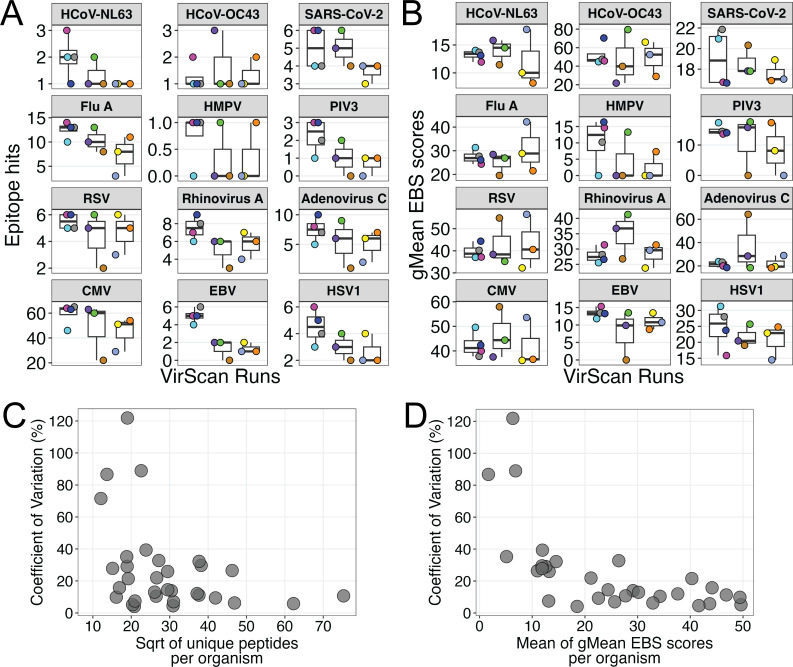
Analysis of antibody responses to clinically relevant viruses and bacteria from a single healthy adult donor demonstrates VirScan inter-assay variability. (**A, B**) Box-and-whisker plots of 10 replicate samples from one donor across three independent VirScan runs assessing reproducibility of (**A**) epitope hits and (**B**) gMean EBS score metrics for select respiratory viruses and herpesviruses. Colors indicate individual replicate samples. Lower and upper hinges are the first and third quartiles, solid line represents the median, and whiskers are 1.5 × interquartile range. (**C**) Scatter plot of coefficient of variation (reported as a percent, %) relative to the square root of the number of unique peptides per organism within the VirScan phage library for select respiratory viruses, herpesviruses, and bacteria. (**D**) Scatter plot of coefficient of variation (%) relative to the mean of the gMean EBS scores per organism for all 10 replicates for select respiratory viruses, herpesviruses, and bacteria. For panels **C and D**, three viruses with no reported coefficient of variation were excluded.

Overall, our data suggest that there is no association between differences in measurements of antibody responses using VirScan and the use of self-sampling or in-clinic blood collection methods. Thus, we investigated whether differences in epitope hits and gMean EBS score metrics seen within paired blood samples could be due to intrinsic inter-assay variability within VirScan. To evaluate VirScan assay variability and reproducibility, we tested serum from one healthy adult donor using 10 replicate samples across three independent assay runs within the same plates and sequencing batches as the experimental samples in this study. We found that while 83% of the 35 pathogens assessed had a range of less than 10 epitope hits, CMV had a range of 43 epitope hits across the 10 replicate samples tested (Table S5), demonstrating that VirScan measurements are subject to variability. However, only 16 of the 35 pathogens (46%) assessed had ranges of less than 20 for gMean EBS scores, while some other pathogens, such as HCoV-OC43, had higher ranges (57.8) or did not have enough data to be properly assessed (HCoV-HKU1 [N5], PIV1, PIV4) (Fig. 5A and B; Table S5). When we interrogated the total epitope hits for the 10 replicate samples, we found 3 of the samples had lower epitope hits for many of the viruses assessed (Fig. 5A). However, these three replicate samples did not have consistently lower virus-specific gMean EBS scores (Fig. 5B). VirScan samples are screened in duplicates, and positive detection of antibody-bound epitopes requires both duplicates to reach a pre-determined EBS score threshold. We identified that two of the three replicate samples had lower overall correlation between technical duplicates compared to the other eight replicate samples (data not shown). Thus, the peptide EBS threshold selection and discordance between technical duplicates likely underlie the lower total epitope hits.

We then used the coefficient of variation formula to evaluate assay reproducibility across the three independent runs and found that almost half (49%) of the 35 clinically relevant pathogens assessed had percentages under 20% variability (Table S5). We also identified that the four pathogens with coefficients of variation over 70% also had lower numbers of unique peptide sequences within the VirScan phage library (Fig. 5C) and lower means of gMean EBS scores (Fig. 5D). Overall, these data suggest that VirScan is subject to inter-assay variability, which may be driven by lower assay sensitivity, antibody detection thresholding decisions, and discordance between technical duplicates. Thus, the differences in VirScan metrics of epitope hits and gMean EBS scores between participant-matched capillary (serum) and venous (plasma) blood samples are more likely due to intrinsic inter-assay variability rather than a technical effect due to blood sample collection method or blood preparation.

## DISCUSSION

The reliance on in-clinic venipuncture blood collection during longitudinal clinical studies remains a logistical barrier to consistent volunteer participation and retention. Providing study participants with more convenient blood collection methods that can be performed outside of the clinic would allow researchers to expand participant outreach and recruitment to geographically diverse areas and increase access to populations that otherwise might not participate in research, to conduct fully remote or hybrid remote/in-person studies, and increase the number of longitudinal sample collection time points without burdening participants to travel to clinical sites. In this study, we assessed historical data of VirScan PhIP-Seq epitope profiling on convenience samples of participant-matched capillary (serum) and venous (plasma) blood samples collected on the same day by Tasso devices and in-clinic venipuncture to assess for differences in pathogen-specific antibody measurements. Overall, we found no association between capillary (serum) or venous (plasma) blood collection method and differences in VirScan epitope hits and gMean EBS scores. However, the differences found in pathogen-specific antibody measurements between paired blood samples are more likely due to intrinsic VirScan inter-assay variability, as shown in the data from our control donor samples.

To investigate whether at-home capillary blood self-sampling devices could reliably replace in-clinic venipunctures for measuring broad antibody responses to human pathogens by VirScan, we assessed the agreement of VirScan-specific antibody measurements for all viral, bacterial, and fungal pathogens included in the phage library. Overall, we found that for organisms with detectable antibody responses in at least one paired blood sample, 95% differed in epitope hits between −3 and 3, and gMean EBS scores ranged between −24.4 and 27.5. As VirScan assay variability and reproducibility have not been well described previously, these ranges in differences of paired sample measurements provide a general baseline to assess antibodies specific to individual pathogens. We next investigated differences in VirScan metrics of paired capillary (serum) and venous (plasma) blood samples for 35 clinically relevant viral and bacterial pathogens individually and in groups. We found that the herpesviruses had larger differences in epitope hits between paired blood samples but had smaller ranges of gMean EBS score differences than the respiratory viruses assessed. These disparate results were possibly driven by the larger ranges in epitope hits across all samples for some herpesviruses, including CMV, EBV, and HSV1, compared to the respiratory viruses assessed. These larger ranges in epitope hits could be due to the larger numbers of unique peptides within the VirScan phage library for herpesviruses compared to most respiratory viruses or differences in virus-specific antibody waning over time. In addition, the herpesviruses had overall stronger concordance between epitope hits and gMean EBS scores between paired blood samples. The respiratory viruses had overall larger differences in gMean EBS scores compared to herpesviruses, which were likely driven by respiratory viruses with weaker measurement concordance and outliers with large differences (>100) in gMean EBS scores. These larger differences in gMean EBS scores among respiratory viruses may also be due to our reporting of percentile ranges for the entire range of gMean EBS scores within each pathogen group observed in our study. Due to the observed heteroscedasticity of gMean EBS score differences, the percentile ranges we reported may be narrower than those for the higher gMean EBS score differences and wider than those for the lower gMean EBS score differences. Most of the clinically relevant pathogens we assessed had moderate to strong concordance of VirScan antibody measurements between participant-matched Tasso (serum) and venipuncture (plasma) samples. In addition, we found no association between blood collection method compared to epitope hits or gMean EBS scores variability for the pathogens we assessed. Together, these data suggest that the variability in epitope hits and gMean EBS scores between paired blood samples is likely due to assay variability rather than technical differences in blood sample collection or processing methods.

We next investigated whether the variability found in epitope hits and gMean EBS scores was due to intrinsic inter-assay variability within VirScan. Currently, there are no reference standards or reproducible positive control samples developed for VirScan, so to evaluate inter-assay reproducibility and variability, we tested serum from one healthy adult donor in 10 technical replicates across three independent VirScan runs within the same plates and sequencing batches as the experimental samples in this study. Overall, of the 35 clinically relevant pathogens we selected for further evaluation, we found almost half had an inter-assay variability less than 20%. However, studies that investigate repetitive testing of control samples over time with PhIP-Seq or other next-generation sequencing assays are limited. One assessment of the reproducibility of RNA-seq analyses across multiple research sites found higher reproducibility of sequence feature detection and differential gene expression calls associated with higher read depth and strongly expressed genes (45). Another study detailed the validation of a next-generation sequencing assay to assess a panel of tumor-specific immune responses and found high reproducibility for the 54 target genes (46). However, the authors found limitations to the assay, such as: (i) a high coefficient of variation (61%) in housekeeping genes analyzed in samples containing less than 10% of target malignant nucleic acids; and (ii) less accuracy in clinical interpretation of mutational burden for samples containing less than 50% neoplastic cells (46). One study assessed concordance of a next-generation sequencing tumor gene panel assay for replicate samples taken from two groups of formalin-fixed paraffin-embedded blocks and found a high discordance of detected variants for both groups, which the authors partially attribute to low DNA quality (47). Prior studies assessing variability of multiplex electrochemiluminescent and enzyme-linked immunosorbent assays described that lower assay precision with coefficient of variation measurements above 20% was driven by lower levels of analyte (48, 49). In this study, we found that organisms with higher inter-assay variability of antibody measurements had lower numbers of library peptide sequences, which suggests that the sensitivity of VirScan to detect antibodies to organisms with smaller peptide libraries may be lower than those with larger libraries. Similar to prior studies, we found that the pathogens with the highest inter-assay variability in antibody detection had the lowest overall average of gMean EBS scores, which demonstrates that those antibody responses may have been close to the limit of detection for VirScan, where assay variability is always high.

While we found that VirScan is subject to inter-assay variability of reproducible measurements of epitope hits and EBS scores, our data suggest that the variability in experimental sample measurements for the respiratory viruses and herpesviruses we assessed was not influenced by capillary (serum) or venous (plasma) blood collection method. In addition, the anomalous participant-matched blood samples with larger differences were not due to delayed processing times for Tasso samples or lower whole blood volumes collected by Tasso devices. Thus, our data suggest that the variability of pathogen-specific epitope hits and gMean EBS scores between participant-matched Tasso (serum) or venipuncture (plasma) blood samples was likely not driven by differences in blood sample collection but instead due to the intrinsic technical limitations of VirScan.

There were limitations to this study. VirScan is a technically intensive PhIP-Seq assay, and as with other next-generation sequencing assays, variability can arise from multiple workflow steps, for example, nucleic acid extraction and input sample quality, availability of reference standards, sequencing read quality, and bioinformatics pipeline analyses (50). While VirScan is not a clinical-grade assay, the development of standardized reference samples (51–53) for VirScan could identify when technical errors occur and may mitigate batch effects from phage libraries and manufacturer reagent lot-to-lot variation. As VirScan data from a completed clinical study was used, we were unable to assess the same blood preparation (i.e., serum or plasma only) from self-collected devices compared to in-clinic venipuncture or serum compared to plasma from the same venipuncture. We were unable to assess the stability of Tasso-collected blood samples for VirScan metrics regarding shipping conditions due to the small number of samples shipped to our facility from participants’ homes. We also did not test the stability of self-collected blood samples with different storage and shipping conditions. The small participant sample size was also a limitation that we were unable to control for in this convenience sample study.

Our data overall suggest that the variability in VirScan-specific antibody measurements is more likely due to inter-assay variability than to differences between the paired Tasso (serum) and venipuncture (plasma) sample collections. However, VirScan’s intrinsic inter-assay variability does limit the ability to thoroughly parse the effects on antibody measurement variability from the Tasso and venipuncture blood sample collection methods. While prior studies comparing self-collected blood samples to venipuncture samples showed strongly correlated results across multiple clinical laboratory and serology tests (7–20), further studies directly comparing antigen-specific antibodies measured by VirScan and by clinically validated serology assays would be valuable to improve understanding of the limitations of reproducible antibody detection by VirScan.

Together, our findings suggest that at-home capillary blood self-sampling devices are a practical alternative to in-clinic venipuncture for assays that require a smaller amount of biological input, such as VirScan. However, to limit the introduction of additional technical variables, researchers should not use self-sampling devices and venipuncture blood collection methods or different blood sample preparations interchangeably within longitudinal studies. These data do not establish whether VirScan should replace clinically validated serology assays for measuring longitudinal changes in antibody levels to specific pathogens, and additional study is needed. VirScan may be particularly appropriate for researchers who want to investigate antibody-based broad pathogen exposure histories either cross-sectionally or longitudinally in specific populations. VirScan is also advantageous for low-volume sample collections within remote or decentralized study settings, for time and cost savings compared to performing multiple serology assays for multiple pathogens, and for the ability to screen for antibody binding to more antigenic epitopes than in serology assays that are more sensitive but limited antigenically.

Our study supports the use of capillary blood self-sampling devices for high-throughput detection assays similar to prior studies from our group that used self-sampling blood devices to investigate host immune transcriptional response kinetics in immunocompetent adult volunteers during acute SARS-CoV-2 infection (54, 55). Overall, this study demonstrates that capillary blood self-sampling devices can reliably be used in place of in-clinic venipuncture sample collection for VirScan serosurvey. In addition, these devices provide a practical cost- and time-effective blood collection method for clinical researchers interested in sampling cross-sectional and longitudinal antibody responses to a library of pathogens using VirScan.

## Data Availability

VirScan organism data are provided as a supplemental data file. Sequencing data have been deposited as FASTQ files to NCBI SRA and are accessible under BioProject accession number PRJNA1440018. Vir3 and CoV library annotations are provided as a supplemental data file.

## SUPPLEMENTAL MATERIAL

Data Set S1 spectrum.02454-25-s0001.xlsx Virscan organism summary data for empirical and control samples.

Data Set S2 spectrum.02454-25-s0002.xlsx Annotations for Vir3 and CoV phage libraries.

Supplemental material spectrum.02454-25-s0003.pdf Fig. S1; Tables S1 to S5.

ASM does not own the copyrights to Supplemental Material that may be linked to, or accessed through, an article. The authors have granted ASM a non-exclusive, world-wide license to publish the Supplemental Material files. Please contact the corresponding author directly for reuse.
