# Supplementary material for: Assessing VirScan serosurvey epitope profiling variability between in-clinic venous blood draw and capillary blood self-sampling device
Source: Microbiol Spectr. 2026 Apr 27;14(6):e02454-25. doi: 10.1128/spectrum.02454-25 (PMC13228006; doi:10.1128/spectrum.02454-25)
Supplement: Supplemental material — Fig. S1; Tables S1 to S5. [file spectrum.02454-25-s0003.pdf]

Supplemental Material

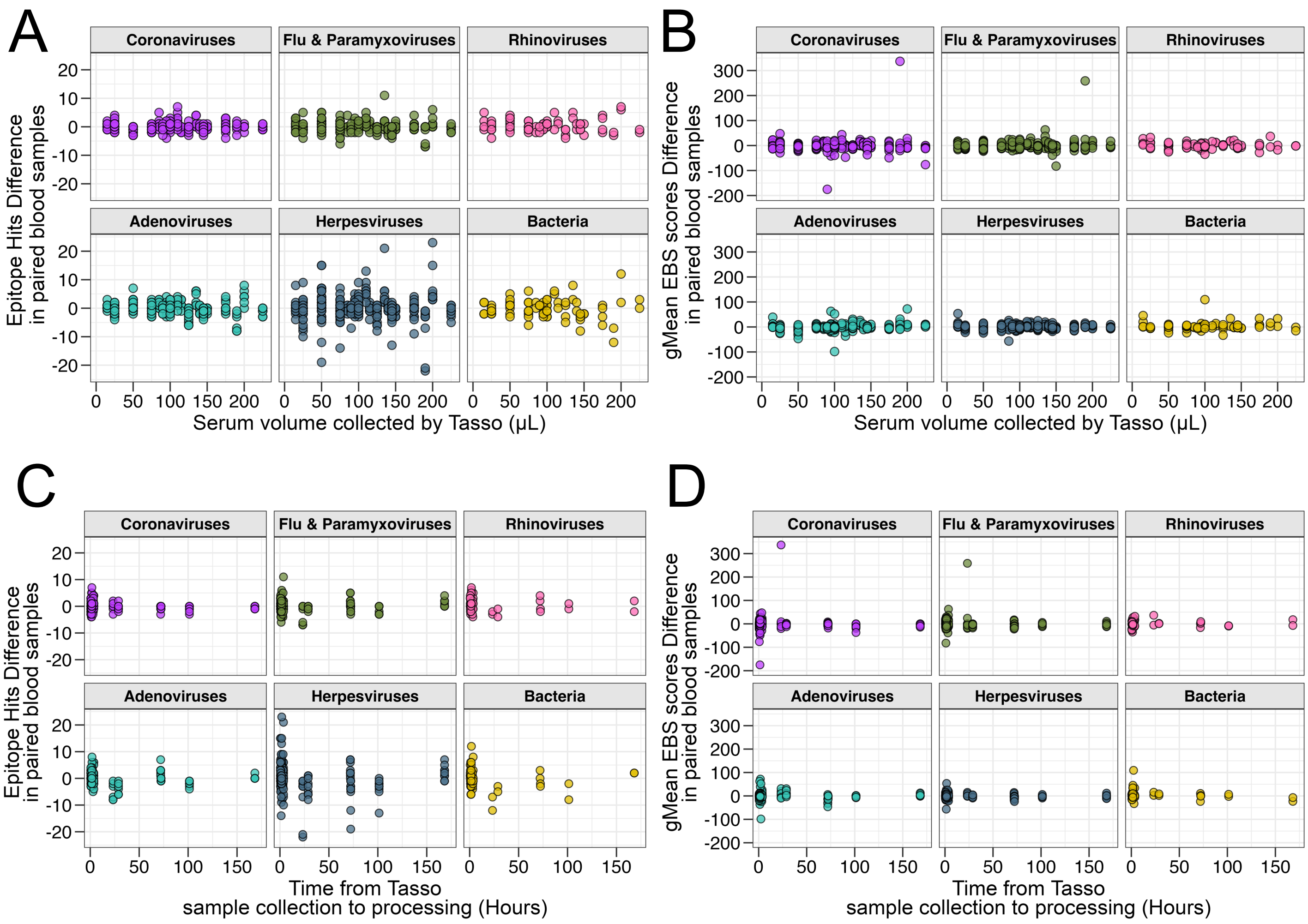

Supplemental Figure 1 (Fig S1). (**A–B**) Scatterplot of differences in (**A**) epitope hits and (**B**) gMean EBS scores of participant-matched Tasso and venipuncture blood samples compared to the volume (μL) of whole blood collected with Tasso device. (**C–D**) Scatterplot of differences in (**C**) epitope hits and (**D**) gMean EBS scores of participant-matched Tasso and venipuncture blood samples compared to the time difference from recorded time of Tasso blood sample collection to recorded time of Tasso blood sample processing in the laboratory.

Supplemental Table 1 (Table S1). Comparison of Bland-Altman analyses of VirScan epitope hits and gMean EBS scores measurements in participant-matched Tasso and venipuncture blood samples for all viruses, bacteria and fungal organisms with and without data from organisms with zero epitope hits in both Tasso and venipuncture participant samples (zero-zero pairs). Column 2 (*n*) is the number of organisms included in the analysis. Column 3 (Mean Difference) is the mean of differences in epitope hits or gMean EBS scores between paired blood samples. Column 4 (2.5th Percentile) is the lower boundary of the 95th percentile center range of measurement differences. Column 5 (97.5th Percentile) is the upper boundary of the 95th percentile center range of measurement differences. Column 6 (Median Difference) is the median of differences in epitope hits or gMean EBS scores between paired blood samples. Column 7 (MAD) is the mean absolute deviation of differences in epitope hits or gMean EBS scores between paired blood samples.

Dataset	*n*	Mean Difference	2.5th Percentile	97.5th Percentile	Median Difference	MAD
**Epitope Hits**						
Including zero–zero pairs	12131	0.011	-2.0	2.0	0.000	0.000
Excluding zero–zero pairs	4705	0.030	-3.0	3.0	0.000	1.000
**gMean EBS Scores**						
Including zero–zero pairs	12131	-0.025	-16.7	17.6	0.000	0.000
Excluding zero–zero pairs	4705	-0.064	-24.4	27.5	-0.567	7.735

Supplemental Table 2 (Table S2). Concordance correlation coefficients with 95% confidence intervals (CIs) comparing VirScan epitope hits and gMean EBS scores measurements in participant-matched Tasso and venipuncture blood samples for clinically-relevant viruses and bacteria for all paired samples.

Organism	Epitope Hits CCC [95% CIs]	gMean EBS CCC [95% CIs]
**Coronaviruses**	**0.69**	**0.49**
HCoV-229E	0.64 [0.46,0.79]	0.73 [0.56,0.82]
HCoV-HKU1	0.43 [0.02,0.75]	0.95 [0.67,0.99]
HCoV-HKU1 (N1)	0.46 [0.2,0.63]	0.45 [0.24,0.79]
HCoV-HKU1 (N2)	0.12 [-0.27,0.5]	0.41 [0.23,0.81]
HCoV-HKU1 (N5)	0.36 [-0.07,1]	0.05 [-0.05,0.96]
HCoV-NL63	0.66 [0.33,0.83]	0.69 [0.56,0.81]
HCoV-OC43	0.46 [0.16,0.67]	0.5 [0.4,0.71]
SARS-CoV-2	0.67 [0.29,0.88]	0.42 [0.07,0.67]
**Influenza & Paramyxoviruses**	**0.89**	**0.63**
Flu A	0.63 [0.37,0.81]	0.23 [0.01,0.62]
Flu B	0.84 [0.68,0.92]	0.21 [0.05,0.83]
HMPV	0.86 [0.69,0.93]	0.91 [0.8,0.97]
PIV1	0.6 [0.26,0.81]	0.87 [0.75,0.91]
PIV2	0.47 [0.11,0.79]	0.86 [0.45,0.97]
PIV3	0.62 [0.4,0.85]	0.69 [0.55,0.84]
PIV4	0.91 [0.84,0.96]	0.76 [0.54,0.91]
RSV	0.58 [0.26,0.78]	0.56 [0.4,0.7]
**Rhinoviruses**	**0.78**	**0.7**
Rhinovirus A	0.8 [0.64,0.93]	0.66 [0.52,0.78]
Rhinovirus B	0.57 [0.21,0.77]	0.71 [0.43,0.88]
**Adenoviruses**	**0.8**	**0.56**
Adenovirus A	0.7 [0.5,0.84]	0.39 [0.23,0.58]
Adenovirus B	0.58 [0.29,0.77]	0.64 [-0.03,0.9]
Adenovirus C	0.73 [0.55,0.86]	0.52 [0.37,0.72]
Adenovirus D	0.62 [0.16,0.84]	0.51 [0.15,0.78]
Adenovirus E	0.75 [0.63,0.85]	0.74 [0.6,0.88]
Adenovirus F	0.75 [0.57,0.87]	0.48 [0.27,0.68]
**Herpesviruses**	**0.95**	**0.8**
HSV1	0.88 [0.77,0.94]	0.75 [0.6,0.88]
HSV2	0.95 [0.91,0.97]	0.69 [0.35,0.8]
VZV	0.78 [0.58,0.87]	0.88 [0.7,0.94]
EBV	0.88 [0.74,0.95]	0.68 [0.47,0.83]
CMV	0.93 [0.83,0.97]	0.71 [0.32,0.93]
HHV6A	0.7 [0.51,0.82]	0.74 [0.63,0.84]
HHV6B	0.81 [0.61,0.9]	0.8 [0.63,0.89]
HHV7	0.69 [0.55,0.84]	0.86 [0.74,0.94]
HHV8	0.8 [0.59,0.91]	0.6 [0.42,0.8]
**Bacteria**	**0.71**	**0.46**
Staph. aureus	0.72 [0.53,0.84]	0.42 [0.27,0.74]
Strep. pneumoniae	0.62 [0.27,0.83]	0.51 [0.17,0.73]

Supplemental Table 3 (Table S3). Concordance correlation coefficients with 95% confidence intervals (CIs) comparing VirScan epitope hit measurements in participant-matched Tasso and venipuncture blood samples for clinically-relevant viruses and bacteria among 34 independent individuals, when the first set of paired measurements was selected per individual. Column 3 (ΔCCC) is the difference in CCC, computed as the CCC among the 34 independent observations (reduced dataset) minus the CCC among all samples (36 paired samples). Columns 4 (ΔLower 95% CI) and 5 (ΔUpper 95% CI) are the differences in the lower 95% CIs and upper 95% CIs between the two datasets (reduced dataset minus all samples). Column 6 (All Interpretation) is the interpretation of the CCC calculated from all. Column 7 (Reduced Interpretation) is the interpretation of the CCC calculated from the reduced dataset.

Organism	Epitope Hits[1] CCC [95% CIs]	ΔCCC	ΔLower 95% CI	ΔUpper 95% CI	All Interpretation	Reduced Interpretation
**Coronaviruses**						
HCoV-229E	0.62 [0.44,0.78]	-0.014	-0.026	-0.008	Moderate	Moderate
HCoV-HKU1	0.41 [0.03,0.76]	-0.025	0.003	0.013	Weak	Weak
HCoV-HKU1 (N1)	0.44 [0.17,0.64]	-0.020	-0.029	0.011	Weak	Weak
HCoV-HKU1 (N2)	0.11 [-0.25,0.53]	-0.010	0.021	0.031	Poor	Poor
HCoV-HKU1 (N5)	0.35 [-0.09,1]	-0.003	-0.014	0.000	Weak	Weak
HCoV-NL63	0.68 [0.35,0.84]	0.016	0.016	0.005	Moderate	Moderate
HCoV-OC43	0.45 [0.15,0.67]	-0.005	-0.014	0.003	Weak	Weak
SARS-CoV-2	0.72 [0.36,0.91]	0.057	0.074	0.031	Moderate	Moderate
**Influenza & Paramyxoviruses**						
Flu A	0.67 [0.42,0.83]	0.032	0.050	0.020	Moderate	Moderate
Flu B	0.87 [0.75,0.93]	0.028	0.069	0.014	Strong	Strong
HMPV	0.89 [0.78,0.95]	0.034	0.092	0.020	Strong	Strong
PIV1	0.58 [0.21,0.78]	-0.027	-0.058	-0.028	Moderate	Moderate
PIV2	0.45 [0.07,0.76]	-0.021	-0.042	-0.030	Weak	Weak
PIV3	0.65 [0.39,0.84]	0.024	-0.006	-0.004	Moderate	Moderate
PIV4	0.91 [0.83,0.95]	-0.003	-0.005	-0.005	Strong	Strong
RSV	0.69 [0.48,0.84]	0.112	0.219	0.061	Moderate	Moderate
**Rhinoviruses**						
Rhinovirus A	0.81 [0.63,0.93]	0.007	-0.004	0.000	Strong	Strong
Rhinovirus B	0.58 [0.21,0.78]	0.009	0.001	0.005	Moderate	Moderate
**Adenoviruses**						
Adenovirus A	0.74 [0.53,0.86]	0.034	0.028	0.020	Moderate	Moderate
Adenovirus B	0.66 [0.44,0.81]	0.082	0.147	0.036	Moderate	Moderate
Adenovirus C	0.77 [0.6,0.88]	0.037	0.043	0.023	Moderate	Moderate
Adenovirus D	0.75 [0.51,0.88]	0.133	0.348	0.044	Moderate	Moderate
Adenovirus E	0.77 [0.64,0.86]	0.015	0.009	0.012	Moderate	Moderate
Adenovirus F	0.75 [0.55,0.88]	-0.001	-0.024	0.008	Moderate	Moderate
**Herpesviruses**						
HSV1	0.88 [0.77,0.95]	0.006	0.009	0.007	Strong	Strong
HSV2	0.95 [0.92,0.98]	0.002	0.007	0.010	Strong	Strong
VZV	0.79 [0.61,0.89]	0.013	0.027	0.014	Moderate	Moderate
EBV	0.91 [0.79,0.96]	0.026	0.044	0.012	Strong	Strong
CMV	0.95 [0.83,0.98]	0.020	0.000	0.011	Strong	Strong
HHV6A	0.7 [0.5,0.82]	0.000	-0.004	0.006	Moderate	Moderate
HHV6B	0.82 [0.64,0.92]	0.014	0.027	0.017	Strong	Strong
HHV7	0.7 [0.56,0.84]	0.013	0.012	0.002	Moderate	Moderate
HHV8	0.8 [0.61,0.92]	0.000	0.022	0.008	Strong	Strong
**Bacteria**						
Staph. aureus	0.76 [0.56,0.87]	0.035	0.032	0.027	Moderate	Moderate
Strep. pneumoniae	0.74 [0.43,0.88]	0.114	0.164	0.051	Moderate	Moderate

[1] 34 paired samples. Samples collected at secondary timepoints from two participants excluded.

Supplemental Table 4 (Table S4). Concordance correlation coefficients with 95% confidence intervals (CIs) comparing VirScan gMean EBS score measurements in participant-matched Tasso and venipuncture blood samples for clinically-relevant viruses and bacteria among 34 independent individuals, when the first set of paired measurements was selected per individual. Column 3 (ΔCCC) is the difference in CCC, computed as the CCC among the 34 independent observations (reduced dataset) minus the CCC among all samples (36 paired samples). Columns 4 (ΔLower 95% CI) and 5 (ΔUpper 95% CI) are the differences in the lower 95% CIs and upper 95% CIs between the two datasets (reduced dataset minus all samples). Column 6 (All Interpretation) is the interpretation of the CCC calculated from all. Column 7 (Reduced Interpretation) is the interpretation of the CCC calculated from the reduced dataset.

Organism	gMean EBS[1] CCC [95% CIs]	ΔCCC	ΔLower 95% CI	ΔUpper 95% CI	All Interpretation	Reduced Interpretation
**Coronaviruses**						
HCoV-229E	0.72 [0.55,0.82]	-0.009	-0.010	0.000	Moderate	Moderate
HCoV-HKU1	0.95 [0.68,1]	0.000	0.004	0.003	Strong	Strong
HCoV-HKU1 (N1)	0.43 [0.23,0.73]	-0.012	-0.014	-0.058	Weak	Weak
HCoV-HKU1 (N2)	0.7 [0.31,0.87]	0.292	0.077	0.065	Weak	Moderate
HCoV-HKU1 (N5)	0.05 [-0.05,0.97]	-0.001	-0.003	0.013	Poor	Poor
HCoV-NL63	0.69 [0.54,0.83]	0.003	-0.017	0.014	Moderate	Moderate
HCoV-OC43	0.51 [0.4,0.74]	0.009	0.004	0.027	Moderate	Moderate
SARS-CoV-2	0.48 [0.1,0.71]	0.060	0.029	0.042	Weak	Weak
**Influenza & Paramyxoviruses**						
Flu A	0.22 [0.01,0.62]	-0.010	0.001	-0.003	Weak	Weak
Flu B	0.8 [0.73,0.87]	0.582	0.681	0.046	Weak	Strong
HMPV	0.92 [0.8,0.97]	0.005	0.008	0.003	Strong	Strong
PIV1	0.88 [0.72,0.91]	0.003	-0.029	0.004	Strong	Strong
PIV2	0.86 [0.43,0.97]	-0.003	-0.021	-0.002	Strong	Strong
PIV3	0.7 [0.54,0.86]	0.007	-0.003	0.020	Moderate	Moderate
PIV4	0.75 [0.51,0.9]	-0.008	-0.031	-0.006	Moderate	Moderate
RSV	0.56 [0.37,0.71]	0.010	-0.023	0.013	Moderate	Moderate
**Rhinoviruses**						
Rhinovirus A	0.68 [0.49,0.8]	0.014	-0.038	0.021	Moderate	Moderate
Rhinovirus B	0.71 [0.42,0.87]	-0.007	-0.001	-0.006	Moderate	Moderate
**Adenoviruses**						
Adenovirus A	0.38 [0.23,0.57]	-0.005	0.001	-0.008	Weak	Weak
Adenovirus B	0.66 [-0.1,0.91]	0.015	-0.071	0.011	Moderate	Moderate
Adenovirus C	0.55 [0.36,0.74]	0.025	-0.009	0.021	Moderate	Moderate
Adenovirus D	0.52 [0.18,0.8]	0.008	0.031	0.021	Moderate	Moderate
Adenovirus E	0.74 [0.6,0.88]	0.000	-0.001	0.003	Moderate	Moderate
Adenovirus F	0.47 [0.23,0.7]	-0.014	-0.038	0.021	Weak	Weak
**Herpesviruses**						
HSV1	0.75 [0.58,0.87]	-0.001	-0.011	-0.002	Moderate	Moderate
HSV2	0.7 [0.35,0.81]	0.010	0.001	0.010	Moderate	Moderate
VZV	0.88 [0.72,0.95]	0.001	0.013	0.006	Strong	Strong
EBV	0.62 [0.39,0.78]	-0.061	-0.074	-0.047	Moderate	Moderate
CMV	0.7 [0.29,0.93]	-0.014	-0.028	0.009	Moderate	Moderate
HHV6A	0.73 [0.62,0.84]	-0.004	-0.006	-0.002	Moderate	Moderate
HHV6B	0.77 [0.6,0.89]	-0.029	-0.035	-0.002	Strong	Moderate
HHV7	0.81 [0.68,0.91]	-0.052	-0.066	-0.032	Strong	Strong
HHV8	0.6 [0.4,0.8]	-0.006	-0.022	0.007	Moderate	Moderate
**Bacteria**						
Staph. aureus	0.42 [0.29,0.73]	-0.005	0.022	-0.007	Weak	Weak
Strep. pneumoniae	0.52 [0.2,0.76]	0.016	0.029	0.031	Moderate	Moderate

[1] 34 paired samples. Samples collected at secondary timepoints from two participants excluded.

Supplemental Table 5 (Table S5). Serum from one healthy adult donor was assessed across three independent runs with a combined 10 replicate samples. Column 2 (Number of Peptides) is the number of unique peptides per organism within the VirScan phage library. Column 3 (Epitope Hits Range) is the range of total epitope hits across 10 replicate samples per organism. Column 4 (gMean EBS scores Range) is the range of gMean EBS scores across 10 replicate samples per organism. Column 5 (Mean of Run Means (EBS)) is the mean of gMean EBS scores across three independent runs. Column 6 (Std Dev of Run Means (EBS)) is the standard deviation of the mean of gMean EBS scores across three independent runs. Column 7 (Coefficient of Variation (%)) is the calculated coefficient of variation (std dev ÷ mean) and reported as a percentage.

Organism	Number of Peptides	Epitope Hits Range	gMean EBS scores Range	Mean of Run Means (EBS)	Std Dev of Run Means (EBS)	Coefficient of Variation (%)
**Coronaviruses**						
HCoV-229E	360	3	18.0	12.3	3.6	29.1
HCoV-HKU1	507	2	15.2	6.5	5.7	88.9
HCoV-HKU1 (N1)	351	2	12.8	5.1	1.8	35.2
HCoV-HKU1 (N2)	357	3	19.6	5.6	6.8	121.8
HCoV-HKU1 (N5)	254	0	0.0	0.0	0.0	NaN
HCoV-NL63	435	2	9.6	13.0	1.0	7.5
HCoV-OC43	410	2	57.8	49.4	2.6	5.2
SARS-CoV-2	434	3	5.2	18.4	0.7	4.0
**Influenza and Paramyxoviruses**						
Flu A	5670	11	22.7	27.7	3.0	10.7
Flu B	962	3	15.1	25.8	1.8	6.9
HMPV	144	1	16.4	5.7	4.1	71.5
PIV1	118	0	0.0	0.0	0.0	NaN
PIV2	184	1	9.6	1.7	1.4	86.7
PIV3	228	3	17.6	11.5	3.2	27.9
PIV4	88	0	0.0	0.0	0.0	NaN
RSV	957	4	24.2	41.9	1.9	4.5
**Rhinoviruses**						
Rhinovirus A	680	6	17.5	30.4	3.9	13.0
Rhinovirus B	259	4	32.5	49.8	4.9	9.8
**Adenoviruses**						
Adenovirus A	874	3	39.0	24.8	3.6	14.4
Adenovirus B	922	7	17.3	28.8	4.0	13.9
Adenovirus C	735	9	45.9	26.9	8.8	32.8
Adenovirus D	874	4	26.6	13.1	3.4	26.0
Adenovirus E	567	4	19.1	11.5	4.5	39.3
Adenovirus F	714	4	34.3	20.7	4.5	21.9
**Herpesviruses**						
HSV1	1763	4	16.8	22.4	2.1	9.3
HSV2	1456	4	18.2	11.5	3.4	29.6
VZV	1383	7	27.4	37.8	4.5	12.0
EBV	2146	6	15.3	10.8	2.8	26.4
CMV	3883	43	21.8	43.7	2.5	5.8
HHV6A	1421	5	89.5	47.2	5.3	11.2
HHV6B	703	7	23.6	34.5	3.6	10.4
HHV7	1420	6	22.2	14.1	4.6	32.2
HHV8	2206	2	51.0	32.9	2.0	6.2
**Bacteria**						
Staph. aureus	371	6	34.9	41.1	8.8	21.5
Strep. pneumoniae	285	12	36.0	44.8	7.1	15.8
